# (Re)Introducing the Gerontology and Geriatrics Curricular Standards and Guidelines in Higher Education

**DOI:** 10.1093/geroni/igab046.2131

**Published:** 2021-12-17

**Authors:** Tamar Shovali, Marilyn Gugliucci, Nina Silverstein

## Abstract

The Academy for Gerontology in Higher Education (AGHE), the education member group of GSA, is the only national institutional membership organization devoted primarily to gerontology and geriatrics education. Its mission provides for development and sponsorship of initiatives to advance the field of aging through its focus on education in gerontology/geriatrics. AGHE first published the Gerontology and Geriatrics Curricular Standards over three decades ago – a document that has been an integral resource for implementing/revising programs in liberal arts, the sciences, and more recently, health professions. To meet the needs in the field for increased breadth and depth of content, the new 7th edition of the educational guidelines fully embraces competency-based education for gerontology, as the health professions programs have for years. Our first presenter will provide an overview of the new edition. The second presenter will focus on associate degree programs in gerontology and their unique contribution to higher education. The third will present on undergraduate programs in gerontology explaining how these programs give students an edge in today’s job market. The fourth presenter will address graduate programs in gerontology, describing master’s degree programs and doctoral degree programs in gerontology and aging studies. The fifth presenter will discuss health professions programs including geriatrics curricula for osteopathic medical education, gerontology/geriatrics curricula for health-related programs and the doctor of pharmacy degree programs. Presentations will provide expert recommendations for program development through mapping AGHE’s Gerontology Competencies for Undergraduate and Graduate Education to programs in higher education. Nina Silverstein will serve as discussant.

